# CTSC Confers Radioresistance in Hepatocellular Carcinoma by Regulating Myeloid‐Derived Suppressor Cells

**DOI:** 10.1111/jcmm.71153

**Published:** 2026-04-21

**Authors:** Jiahuan Xu, Bilin Zhang, Shirui Yang, Shaoran Song, Jie Wu, Yunzhi Dang

**Affiliations:** ^1^ Department of Radiation Oncology Shaanxi Provincial People's Hospital Xi'an Shaanxi China; ^2^ Xi'an Medical University Xi'an Shaanxi China; ^3^ Medical School of Yan'an University Yan'an Shaanxi China; ^4^ State Key Laboratory of Cancer Biology Fourth Military Medical University Xi'an Shaanxi China

**Keywords:** CTSC, CXCL1, hepatocellular carcinoma, myeloid‐derived suppressor cells, radioresistance

## Abstract

Radiotherapy is an important therapeutic modality for advanced hepatocellular carcinoma (HCC), but the limited understanding of radioresistance mechanisms in HCC has hindered its further clinical development. This work aimed to clarify the essential role of Cathepsin C (CTSC) in regulating radioresistance in HCC. The expression of CTSC in HCC tissues was analysed using real‐time PCR (RT‐PCR) and immunohistochemistry. The role of CTSC in radiotherapy resistance of HCC was investigated through in vitro experiments, in vivo studies (subcutaneous and orthotopic liver tumour models) and clinical data analysis. Additionally, in vivo experiments were conducted to evaluate the effect of blocking the CTSC signalling pathway on reversing radiotherapy resistance in HCC. CTSC expression was significantly higher in HCC tissues than in adjacent non‐tumour tissues. Correlation analysis showed that positive CTSC expression was positively associated with aggressive clinicopathological features, including increased tumour number, large tumour size, absence of tumour encapsulation, microvascular invasion and advanced TNM stage. Survival analysis further revealed that CTSC overexpression was linked to poorer overall survival (OS) and progression‐free survival (PFS) in HCC patients. In vitro experiments demonstrated that CTSC overexpression increased the clonogenic survival rate of Huh7 cells after ionising radiation (IR) and reduced the apoptosis rate. In both subcutaneous and orthotopic liver tumour models, upregulated CTSC expression significantly decreased radiotherapy sensitivity. Clinically, CTSC overexpression was significantly associated with a poor response to radiotherapy. Mechanistically, CTSC promoted the infiltration of myeloid‐derived suppressor cells (MDSCs) while reducing CD8^+^ T cell infiltration by upregulating CXCL1 expression. Importantly, the combination of the CTSC inhibitor AZD7986 with radiotherapy significantly improved radiotherapy sensitivity in HCC models. CTSC contributes to radiotherapy resistance in HCC by recruiting MDSCs. The synergistic application of a CTSC inhibitor with radiotherapy represents an effective combinational treatment strategy for CTSC‐positive HCC.

**Abbreviations**
BLI: bioluminescent imaging
CTSC: Cathepsin C
HCC: hepatocellular carcinoma
HE: haematoxylin and eosin
IHC: immunohistochemistry
IR: ionising radiation
MDSCs: myeloid‐derived suppressor cells

## 1 Introduction

Hepatocellular carcinoma (HCC) is a malignant tumour with high incidence and mortality rates worldwide, characterised by a poor prognosis [1]. Owing to the lack of reliable early diagnostic biomarkers, only a small proportion of HCC patients are eligible for surgical resection [2]. The advent of systemic therapeutic options – such as sorafenib, lenvatinib and the combination of atezolizumab with bevacizumab – has improved outcomes for advanced HCC [3, 4]. However, the frequent development of drug resistance limits the efficacy of these treatments, rendering HCC a persistent clinical challenge. In recent years, advances in radiotherapy techniques, including intensity‐modulated radiotherapy and stereotactic body radiotherapy, have expanded its role in treating advanced HCC [5, 6, 7, 8]. Nevertheless, intrinsic and acquired radioresistance in HCC results in suboptimal therapeutic outcomes, with most patients eventually experiencing local treatment failure or distant metastasis [9, 10]. Thus, a deeper understanding of the molecular mechanisms underlying radioresistance is critical to identifying novel insights and intervention strategies for effective HCC management.

Cathepsin C (CTSC), also known as dipeptidyl peptidase I, is a lysosomal cysteine protease. As a major lysosomal acid hydrolase, CTSC activates various serine proteases, including proteinase 3, neutrophil elastase, cathepsin G, granzymes A/B/C and mast cell chymase [11, 12]. Dysfunction of CTSC is linked to several inflammatory conditions, such as Wegener's granulomatosis, rheumatoid arthritis, pneumonia and viral infections. Accumulating evidence indicates that CTSC plays a significant role in cancer progression; its upregulation modulates the secretion of chemokines and cytokines, thereby shaping the tumour microenvironment [13]. CTSC has also been identified as an oncogene that promotes the progression of multiple cancers, including breast cancer [14], colorectal cancer [15] and HCC. In HCC, specifically, CTSC expression is significantly elevated, and its overexpression enhances cancer cell promotion and metastasis [16]. Notably, the relationship between CTSC and radiosensitivity, whether in HCC or other tumour types, remains unexplored.

Myeloid‐derived suppressor cells (MDSCs) are a heterogeneous population of myeloid progenitor cells that migrate from the bone marrow to tumour sites. In malignant tumours, MDSCs act as key immunosuppressive cells [17]. Both preclinical and clinical studies have shown that MDSC infiltration contributes to radioresistance and resistance to immune checkpoint inhibitors [18]. Conventional fractionated and hypofractionated radiotherapy both increase MDSC numbers [19, 20], which in turn promote tumour radioresistance [21]. Clinical investigations have further revealed that radiotherapy induces MDSC infiltration and immune suppression [22, 23], which are strongly associated with poor patient prognosis [24, 25, 26]. MDSCs represent promising targets for improving radiotherapy efficacy, as supported by studies using pharmacological interventions or genetic approaches [19]. However, the mechanisms by which MDSCs regulate tumour progression and radioresistance remain incompletely understood, and their further elucidation is essential for enhancing radiotherapy outcomes.

In this work, we found that CTSC is overexpressed in HCC and correlates with poor prognosis. In vitro and in vivo experiments demonstrated that CTSC overexpression promotes radioresistance in HCC by recruiting MDSCs. Moreover, combining CTSC inhibition with radiotherapy significantly enhanced therapeutic efficacy in HCC models, indicating that CTSC plays a critical role in mediating radioresistance in HCC.

## 2 Materials and Methods

### 2.1 Immunohistochemistry and Patient Follow‐Up

For immunohistochemical analysis, HCC specimens and their corresponding adjacent tissues were subjected to staining for CTSC using a specific antibody (ab199109, Abcam). In mouse specimens, immunohistochemical staining was performed to detect the expression of CD11b (ab6640, Abcam) and CD8^+^ T cells (ab4055, Abcam).

Immunohistochemical analysis was performed on 4 μm thick paraffin‐embedded tissue sections following standard protocols as described in our previous study. Briefly, tissue sections were mounted on slides and baked at 60°C for 1 h. They were then deparaffinised in xylene and rehydrated through graded ethanol series. Endogenous peroxidase activity was quenched by incubating sections in 3% (v/v) hydrogen peroxide in methanol for 12 min, followed by three washes with phosphate‐buffered saline (PBS). Antigen retrieval was performed by immersing slides in 0.01 mol/L citrate buffer (pH 6.0) and heating in a microwave oven for 30 min. After washing with PBS, sections were incubated overnight at 4°C in a humidified chamber with primary antibodies diluted in PBS containing 1% (w/v) bovine serum albumin. Negative controls were performed by replacing the primary antibody with pre‐immunised mouse serum. Following three 5 min washes with PBS, sections were incubated with peroxidase‐conjugated second antibody (Santa Cruz) for 30 min at room temperature, then washed three times with PBS for 5 min each. The reaction product was visualised with diaminobenzidine for 2 min. Images were captured using a light microscope (Olympus, Japan) equipped with a DP70 digital camera.

Analyses were performed by two independent observers who were blinded to the clinical outcome. The immunostaining intensity was scored on a scale of 0–3:0 (negative), 1 (weak), 2 (medium), or 3 (strong). The percentage of positive cells was evaluated on a scale of 0–4:0 (negative), 1 (1%–25%), 2 (26%–50%), 3 (51%–75%) or 4 (76%–100%). The final immuno‐activity scores were calculated by multiplying the above two scores, resulting in an overall score of 0–12. Each case was ultimately considered ‘negative’ if the final score ranges from 0 to 3 and ‘positive’ if the final score ranges from 4 to 12.

### 2.2 Animal Experiment

All animal experiments were approved by the Animal Ethics Committee of Shaanxi Provincial People's Hospital. Animal experiments were performed using the RS‐200 (RS‐2000 XE Biological Irradiator, RAD.SOURCE) with a dose rate of 1.24 Gy/min. The radiotherapy dose was 8 Gy per session, administered over three consecutive days [27, 28, 29]. During radiotherapy, only the tumour area was exposed to radiation, while the rest of the body was shielded with lead.

Model 1: Subcutaneous Tumour Model. Briefly, luciferase‐labelled Hepa1‐6 cells (4.0 × 10^6^) were inoculated subcutaneously into the right flanks of C57BL/C mice. After 4 weeks, when the tumour diameter reached 8 mm, radiotherapy was initiated. Two weeks later, the mice were euthanised, and the tumours were excised for measurement, weighing and pathological analysis.

Model 2: Orthotopic Liver Tumour Model. Briefly, mice were anaesthetised and the skin was disinfected. After anaesthesia with 7% chloral hydrate, luciferase labelled mouse Hepa1‐6 cells (4.0 × 10^6^) in the 100 μL of PBS mixed with 100 μL Matrigel were injected into the right lobes of livers of the C57BL/6 mice under anaesthesia (10 for each group). The injection site was pressed with a sterile cotton ball for 2 min, and the skin was sutured. The entire procedure was performed under sterile conditions to prevent infection‐related mortality in C57BL/6 mice. After 4 weeks, radiotherapy was administered at a dose of 4 Gy for three consecutive days. Prior to radiotherapy, the location of the orthotopic liver tumour was marked on the corresponding skin. Only the tumour area was irradiated, while non‐tumour areas were shielded with lead. Mice were sacrificed after 2 weeks, and then livers were removed, weighed and subjected to pathological analysis.

### 2.3 Statistical Analysis

Data analysis was performed using SPSS software (version 20.0). For categorical variables, the *χ* ^2^ test was used to calculate *p*‐values, while for quantitative data, the *t*‐test was used. Recurrence and survival data were calculated using the Kaplan–Meier method. Differences were considered statistically significant when *p* < 0.05.

## 3 Results

### 3.1 CTSC Expression is Elevated in HCC and Correlates with Poor Prognosis

**FIGURE 1:**
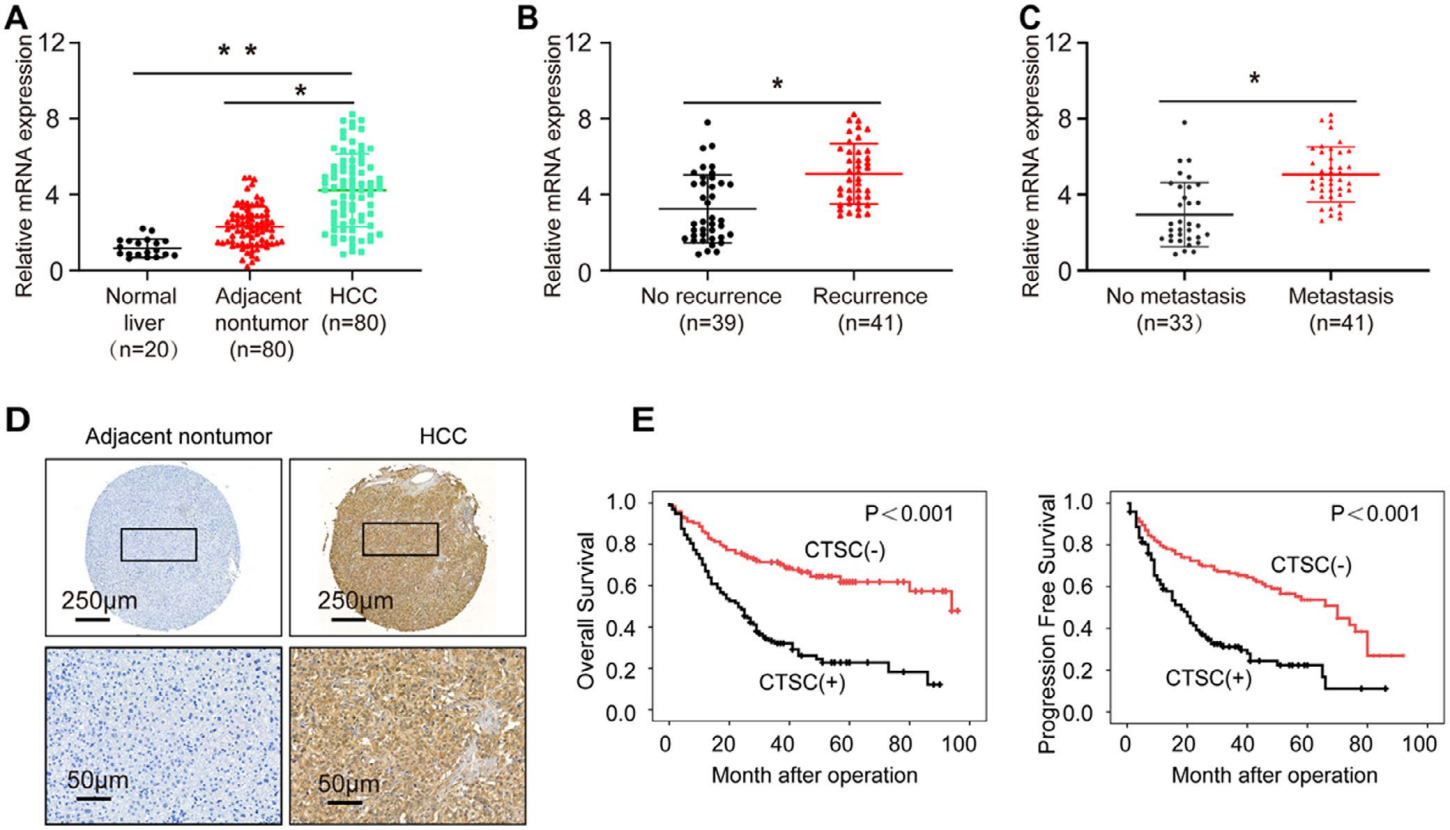
CTSC expression is elevated in HCC and is associated with poor prognosis. (A) RT‐qPCR analysis of CTSC mRNA expression in 20 normal liver tissues and 80 paired adjacent non‐tumour and HCC tissues. (B) Relative CTSC mRNA expression in HCC patients with and without recurrence. (C) Relative CTSC mRNA expression in HCC patients with and without metastasis. (D) Representative IHC images of CTSC protein expression in adjacent non‐tumour and HCC tissues. (E) Kaplan–Meier analysis showing the correlation between CTSC expression and poor overall survival and progression‐free survival in HCC patients. **p < 0.001, *p < 0.05.

To investigate the role of CTSC in HCC, we first detected CTSC expression levels in 20 normal liver tissues and 80 paired HCC and adjacent non‐tumour tissues using RT‐PCR. Results showed that CTSC mRNA expression was significantly higher in HCC tissues compared to both adjacent non‐tumour and normal liver tissues (*p* < 0.05; Figure 1A). Further subgroup analysis revealed that CTSC expression was significantly elevated in HCC patients with recurrence and metastasis compared to those without recurrence and metastasis (Figure 1B,C). IHC staining of a 220‐case HCC tissue microarray demonstrated that CTSC was primarily localised in the cytoplasm, with significantly higher expression in HCC tissues than in adjacent non‐tumour tissues (Figure 1D). Correlation analysis indicated that positive CTSC expression was significantly associated with multiple aggressive clinicopathological features, including increased tumour number, larger tumour size, absence of encapsulation, presence of microvascular invasion and advanced TNM stage (Table 1). Survival analysis revealed that CTSC overexpression was associated with poor OS and PFS in HCC patients (Figure 1E). These results indicate that CTSC is overexpressed in HCC and is associated with poor prognosis.

**TABLE 1:** Correlation between CTSC expression and clinicopathological characteristics in HCC cohort.

Clinicopathological variables		Tumour CTSC expression		*p*
		Negative (*n* = 123)	Positive (*n* = 97)	
Age	≤ 50	28	17	0.339
	> 50	95	80	
Sex	Female	22	9	0.068
	Male	101	88	
Serum AFP	≤ 20 ng/mL	35	28	0.947
	> 20 ng/mL	88	69	
Virus infection	HBV	88	69	0.707
	HCV	12	7	
	HBV + HCV	5	7	
	None	18	14	
Cirrrhosis	Absent	30	25	0.814
	Present	93	72	
Child‐pugh score	Class A	97	79	0.635
	Class B	26	18	
Tumour number	Single	94	62	0.043
	Multiple	29	35	
Maximal tumour size	≤ 5 cm	69	30	< 0.001
	> 5 cm	54	67	
Tumour encapsulation	Absent	88	56	0.032
	Present	35	41	
Microvascular invasion	Absent	40	56	< 0.001
	Present	83	41	
Tumour differentiation	I‐II	88	69	0.947
	III‐IV	35	28	
TNM stage	I‐II	94	57	0.005
	III	29	40	

### 3.2 CTSC Overexpression Promotes HCC Metastasis

To clarify the functional role of CTSC in HCC, we performed in vitro and in vivo experiments to assess its impact on HCC invasion and metastasis. Transwell assay results revealed that upregulating CTSC expression did not enhance the invasive or migratory capacities of Huh7 cells. Similarly, downregulating CTSC expression in nude mice did not significantly affect the metastatic potential of Huh7 cells. These observations suggest that CTSC has no significant effect on HCC metastasis in immunodeficient nude mice (Figure S1).

**FIGURE 2:**
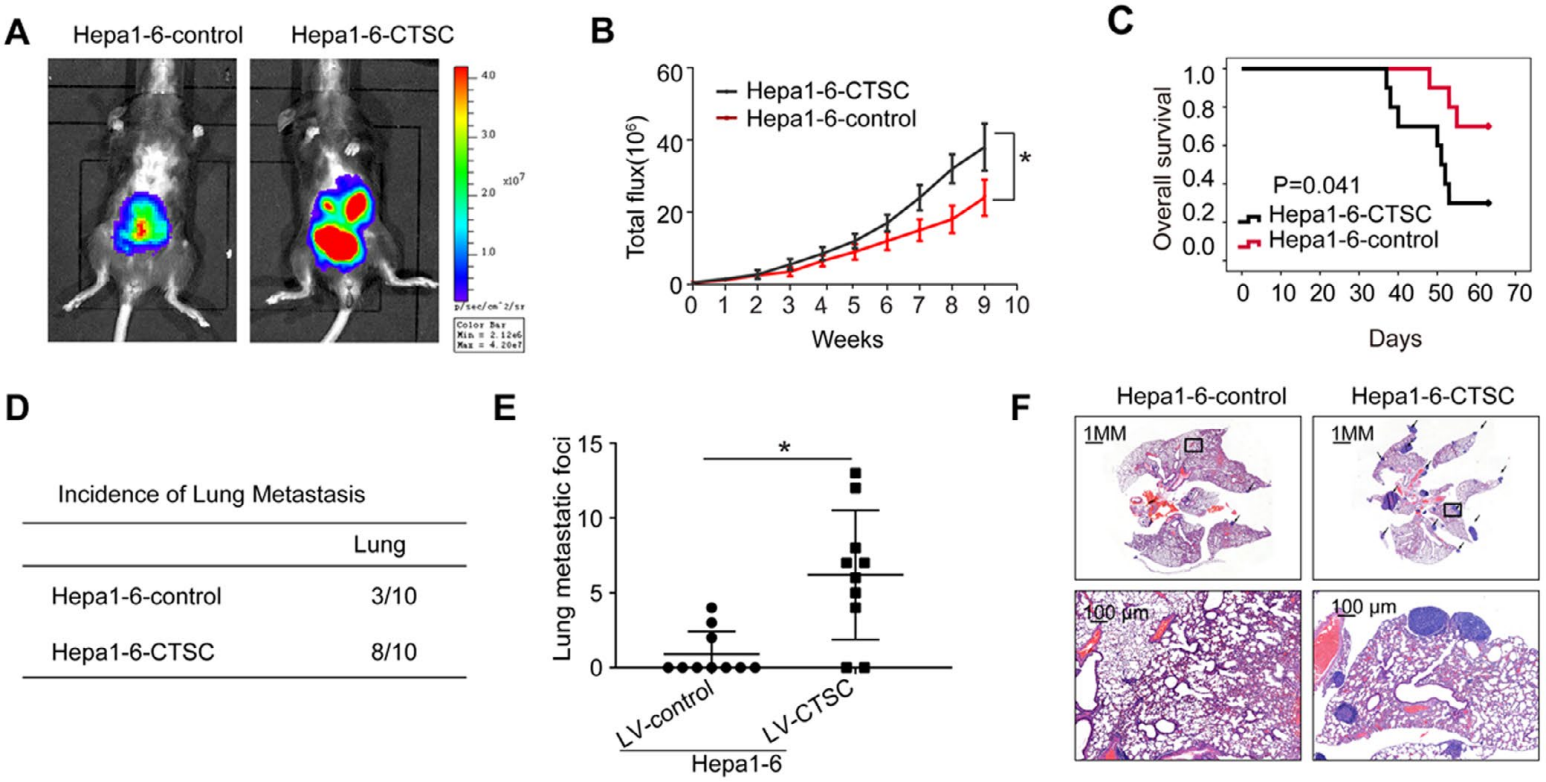
CTSC overexpression promotes HCC metastasis. (A) Bioluminescence imaging showing metastasis in mice. (B) Signal intensity of bioluminescence imaging of indicated group. (C) Overall survival time of mice in both groups. (D) Incidence of lung metastasis in both groups. (E) Number of lung metastatic nodules in both groups. (F) HE staining was applied to exhibit metastatic lung nodules. * *p* < 0.05, by Student's *t* test.

HCC frequently develops in the context of persistent hepatic inflammation and immune system dysregulation. We therefore hypothesised that CTSC might accelerate HCC metastasis by altering the immunological microenvironment. To test this, we stably upregulated CTSC expression in Hepa1‐6 cells via lentiviral transduction, successfully establishing Hepa1‐6‐CTSC cells. Using an intrahepatic orthotopic tumour implantation model in C57BL/6 mice, we further explored the role of CTSC in HCC metastasis. Bioluminescence imaging revealed significantly increased signal intensity in the Hepa1‐6‐CTSC group (Figure 2A,B). Haematoxylin and eosin (HE) staining confirmed a higher incidence of lung metastasis and an increased number of lung nodules in the Hepa1‐6‐CTSC group (Figure 2D–F). Additionally, CTSC overexpression shortened the overall survival rate in C57BL/6 mice (Figure 2C). Collectively, these findings indicate that CTSC overexpression plays a critical role in promoting HCC metastasis.

### 3.3 CTSC Overexpression Enhances Radiotherapy Resistance In Vitro and In Vivo and is Negatively Correlated with Clinical Radiosensitivity

**FIGURE 3:**
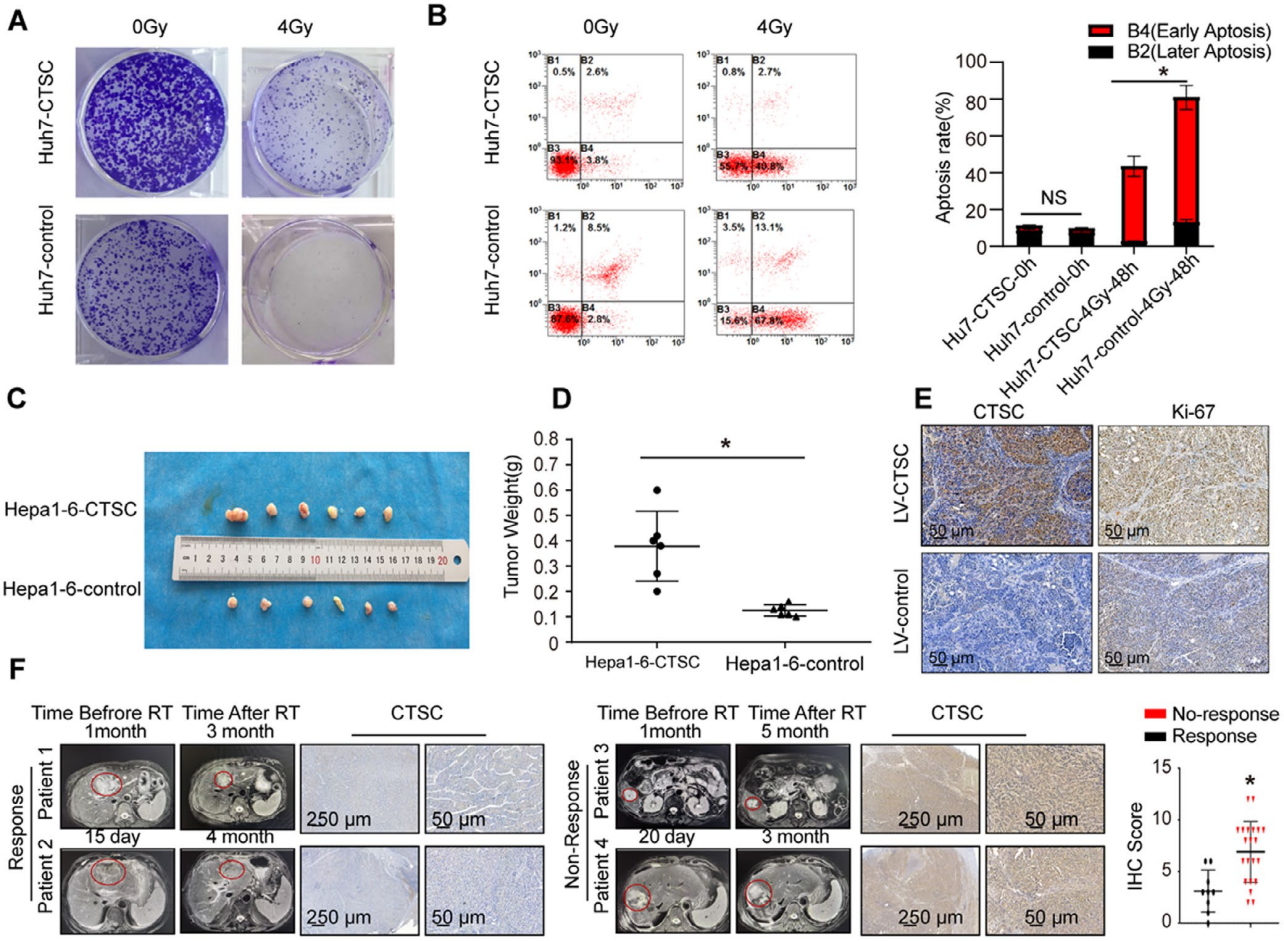
CTSC overexpression enhances radiotherapy resistance in vitro and in vivo and is negatively correlated with clinical radiosensitivity. (A) Clonogenic survival capability of Huh7 cells in both groups after 0 and 4 Gy IR. (B) Apoptosis rate, as well as early and late apoptosis rates of Huh7 cells in both groups 48 h after 0 and 4 Gy radiotherapy. (C) Photographs of indicated xenograft tumours at the end of the experiment (*n* = 6 for each group). (D) Tumour weight in both groups. (E) Ki‐67 and CTSC expression in tumours from both groups. (F) Representative IHC images of HCC specimens from patients who underwent surgical resection before radiotherapy, along with matched pre‐ and post‐radiotherapy MRI images; IHC score of CTSC in the indicated group. * *p* < 0.05, by Student's *t* test.

To further investigate the effect of CTSC on HCC radiosensitivity, we established a stable Huh7‐CTSC cell line using lentiviral transfection and analysed their clonogenic survival capability post IR. The results showed that CTSC overexpression significantly increased the clonogenic survival rate of Huh7 cells after radiotherapy. Moreover, CTSC overexpression markedly reduced the apoptosis rate in both Huh7 cells following IR (Figure 3A,B).

To further assess the impact of CTSC on radiosensitivity, we established a subcutaneous tumour model. Consistent with the in vitro findings, under radiotherapy conditions, the tumour volume and weight in the CTSC overexpression group were significantly higher than those in the control group (Figure 3C,D). Additionally, Ki‐67, a proliferation marker, was expressed at higher levels in CTSC‐overexpressing xenograft tumours after 24 Gy of radiotherapy (Figure 3E). We further analysed the correlation between CTSC expression and radiosensitivity in clinical samples. CTSC expression was detected by IHC in 30 pre‐radiotherapy HCC specimens. The results revealed that high expression of CTSC was associated with poor response to RT, while samples from responders tended to show a reduction in protein levels of CTSC in tumour cells (Figure 3F). In summary, CTSC overexpression is a critical prognostic marker for radiotherapy resistance in HCC.

### 3.4 Depletion of CTSC Expression Enhances HCC Radiosensitivity In Vivo by Decreasing MDSC Infiltration

**FIGURE 4:**
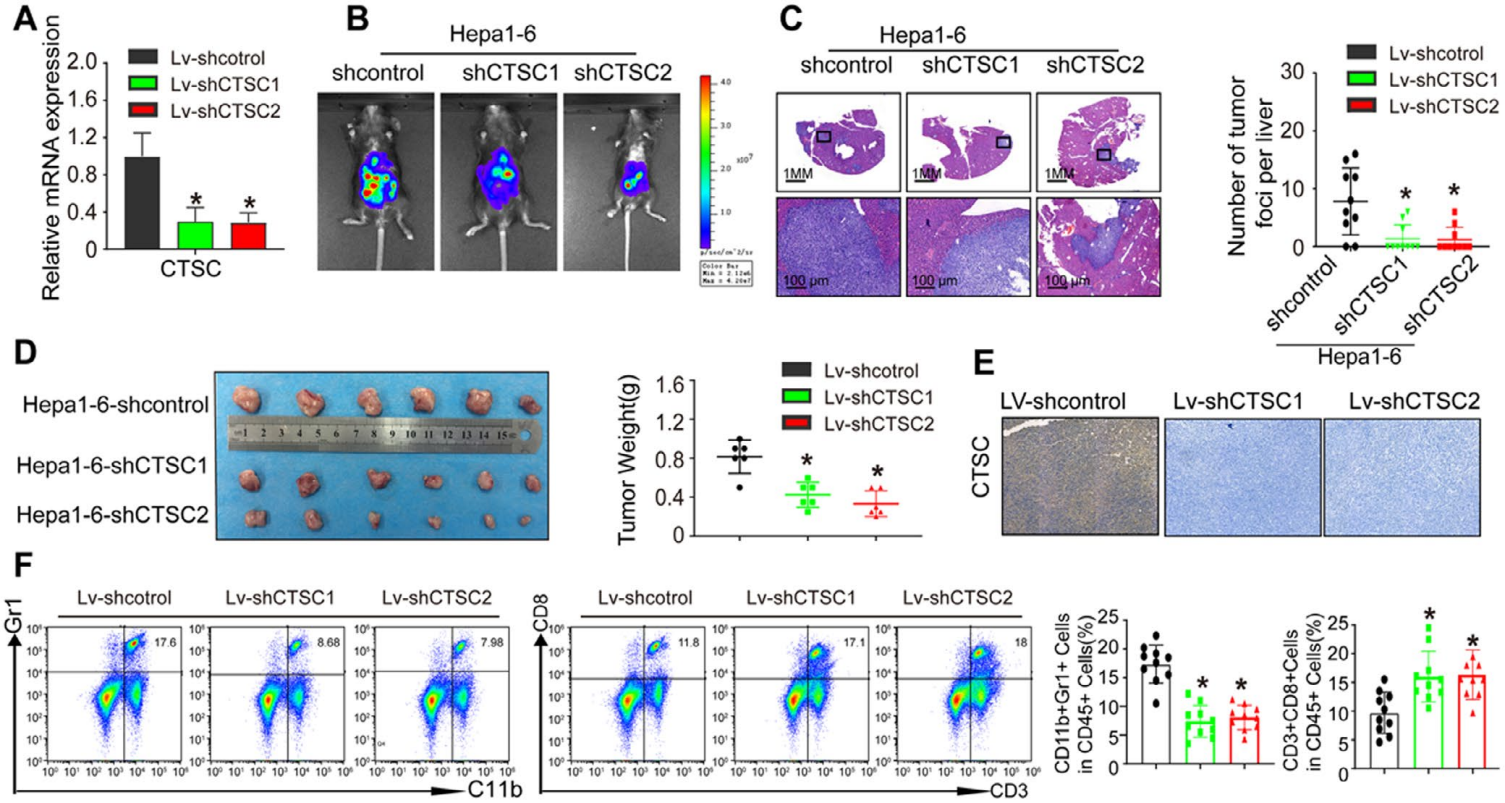
Depletion of CTSC expression enhances HCC radiosensitivity in vivo by decreasing MDSC infiltration. (A) CTSC expression in the indicated cell by Real‐time PCR analysis. (B) Bioluminescence imaging showing metastasis in indicated groups. (C) HE staining showing liver metastatic nodules and the number of liver metastatic nodules in indicated groups 2 weeks after radiation. (D) Photographs of indicated xenograft tumours at the end of the experiment. (E) Tumour weight in both groups. (F) Flow cytometry analysis of MDSC and CD8^+^ T cell infiltration in indicated groups. *p < 0.05, by Student's *t* test.

Persistent liver inflammation and immune cell dysregulation are common aetiological factors in HCC. We therefore hypothesised that CTSC promotes radiotherapy resistance in HCC by modulating the immune microenvironment. We established a luciferase labelled Hepa1‐6‐shCTSC cell line via lentiviral transfection and developed an orthotopic liver tumour model. Specifically, we stably repressed CTSC expression in Hepa1‐6 cells using two independent shRNA (shCTSC‐1 and shCTSC‐2). The expression of CTSC in Hepa1‐6 cell lines was analysed by RT‐PCR (Figure 4A). As shCTSC‐1 and shCTSC‐2 can knock down CTSC expression significantly, we used both Hepa1‐6‐shCTSC‐1 and Hepa1‐6‐shCTSC‐2 to conduct in vivo study. Bioluminescence imaging revealed that tumour burden was significantly reduced in both Lenti‐shCTSC‐1 and Lenti‐shCTSC‐2 knockdown groups (Figure 4B). HE staining demonstrated that CTSC knockdown decreased the number and size of liver tumours (Figure 4C).

To further assess the impact of CTSC on radiosensitivity, we established a subcutaneous tumour model. Consistent with the orthotopic liver tumour model, under radiotherapy conditions, the tumour volume and weight in the Hepa1‐6‐shCTSC‐1 and Hepa1‐6‐shCTSC‐2 groups were significantly lower than those in the control group (Figure 4D). Additionally, CTSC expression was at lower levels in both Lenti‐shCTSC‐1 and Lenti‐shCTSC‐2 knockdown group xenograft tumours after 24 Gy of radiotherapy (Figure 4E). To further elucidate the mechanism by which CTSC promotes radiotherapy resistance in HCC, we analysed the expression of immune cells within liver tumours. Flow cytometry showed that MDSC infiltration was significantly reduced, while CD8^+^ T cell infiltration was markedly increased in both the Hepa1‐6‐shCTSC1 and Hepa1‐6‐shCTSC2 groups (Figure 4F).

### 3.5 CTSC Overexpression Recruits MDSCs via the CTSC‐CXCL1 Signalling Pathway in HCC

**FIGURE 5:**
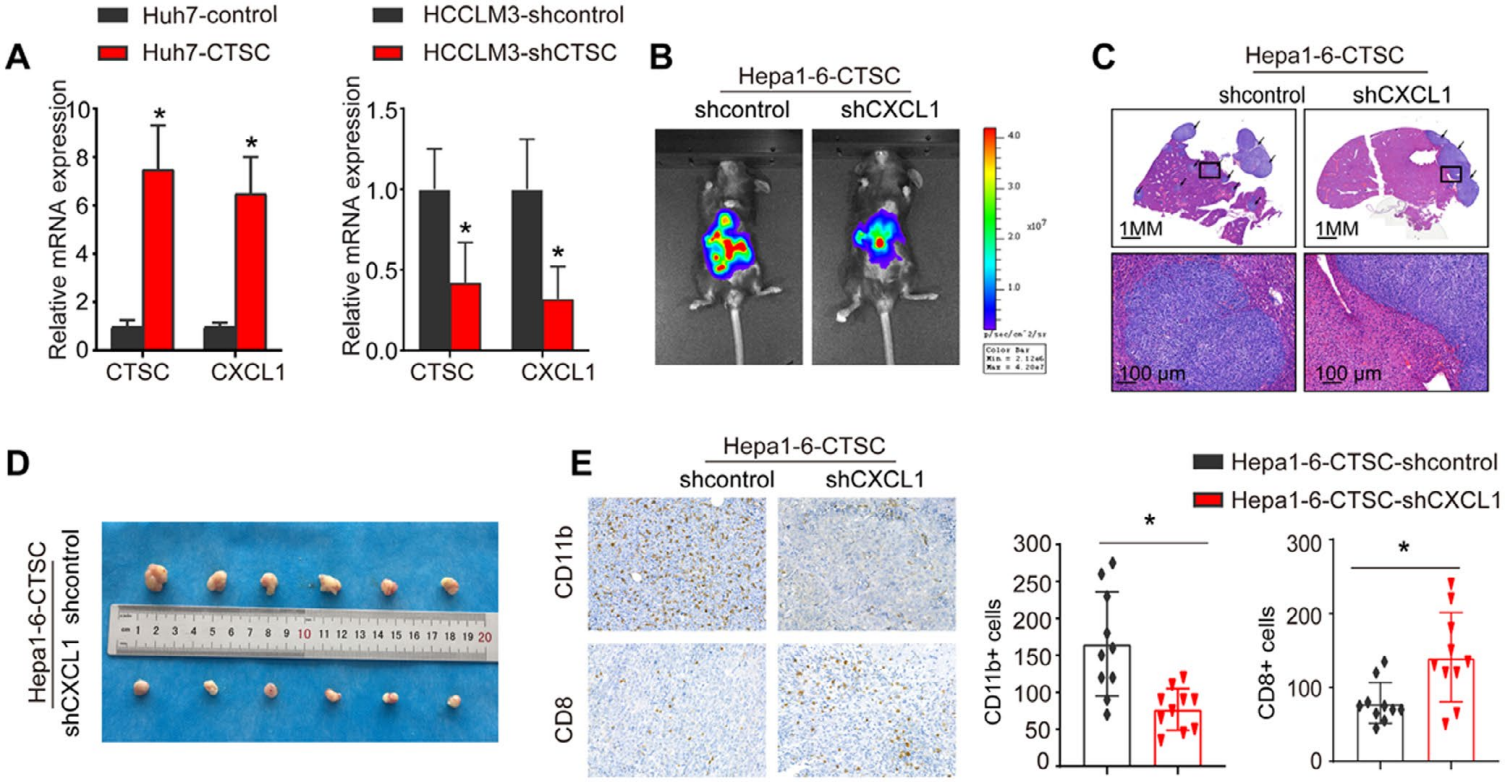
CTSC overexpression recruits MDSCs via the CTSC‐CXCL1 signalling pathway in HCC. (A) Real‐time PCR analysis of CTSC and CXCL1 expression in corresponding cells. (B) Bioluminescence images showing metastasis in both groups. (C) HE staining analysis of liver tumours in both groups 2 weeks after radiotherapy. (D) Photographs of indicated xenograft tumours at the end of the experiment. (E) IHC analysis of MDSC and CD8^+^ T cell infiltration in both groups. *p < 0.05.

To investigate the mechanism by which CTSC recruits MDSCs, we established a Huh7‐CTSC cell line using lentiviral transfection. Transcriptomic differences between Huh7‐control and Huh7‐CTSC cells were analysed using the Affymetrix PrimeView Human Gene Expression Array. Results showed that CTSC overexpression upregulated the expression of several metastasis‐related genes, including CXCL1, ICAM, SLC7A11 and CXCR2 (Table S1). Notably, CXCL1, by binding to its receptor CXCR2, recruits MDSCs, and previous studies have shown that CXCL1 promotes HCC metastasis by recruiting MDSCs [30]. Given the critical role of CXCL1 in tumour progression, we further investigated its function. Real‐time PCR assays demonstrated that CTSC overexpression increased CXCL1 expression in Huh7 and HCCLM3 cells, whereas CTSC knockdown reduced CXCL1 expression (Figure 5A). To explore the role of CXCL1 in HCC radiotherapy resistance, we knocked down CXCL1 expression in Hepa1‐6‐CTSC cells using lentiviral transfection and established an orthotopic liver tumour model in C57BL/C mice. In this model, CXCL1 knockdown significantly reduced tumour burden after IR (Figure 5B,C).

In the subcutaneous tumour model, CXCL1 knockdown markedly enhanced the radiosensitivity of Hepa1‐6‐CTSC cells (Figure 5D). Additionally, IHC revealed that CXCL1 knockdown significantly reduced MDSC infiltration and increased CD8^+^ T cell infiltration (Figure 5E). These results collectively indicate that CTSC promotes radiotherapy resistance in HCC by recruiting MDSCs through the CTSC‐CXCL1 signalling pathway.

### 3.6 Pharmacological Inhibition of the CTSC Signalling Pathway Significantly Enhances HCC Radiosensitivity

**FIGURE 6:**
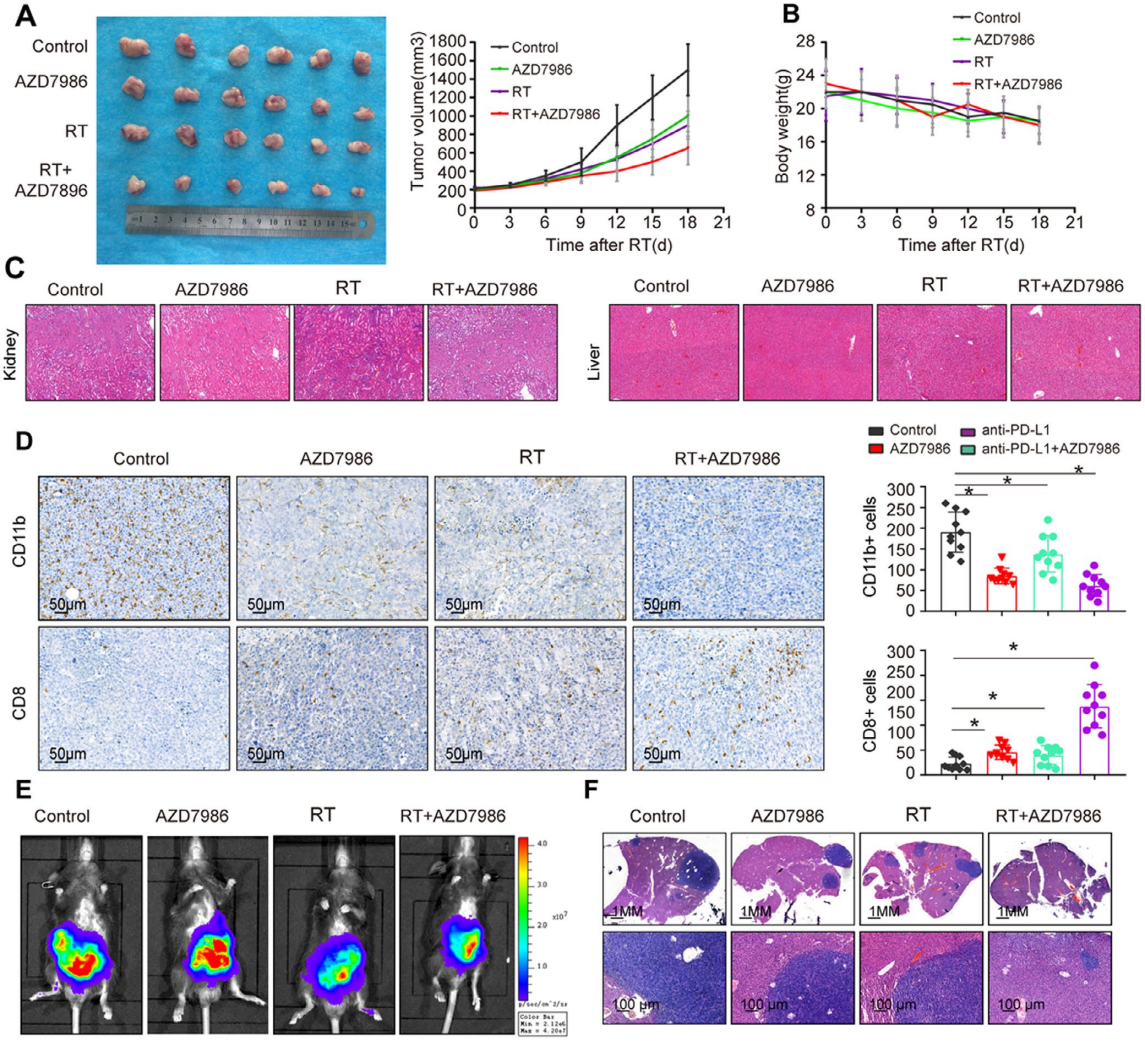
Pharmacological inhibition of the CTSC signalling pathway significantly enhances HCC radiosensitivity. (A–C). C57BL/C mice were subcutaneously injected with 1 × 10^6^ Hepa1‐6 cells. One week later, mice were divided into four groups and treated with control, radiotherapy, AZD7986, RT or RT and AZD7986. In vivo experiments showed that the combination of AZD7986 and radiotherapy significantly inhibited tumour growth (A). Tumour size and volume in the four groups; (B). Body weight of mice in the four groups; (C). IHC analysis of MDSC and CD8^+^ T cell infiltration in the four groups. (D, E). Further analysis of the efficacy of AZD7986 in enhancing IR using an orthotopic liver tumour model. (D). Bioluminescence images were captured for each group; (E). HE staining analysis of tumour metastasis in the four groups 2 weeks after treatment.*p < 0.05, by Student's *t* test.

Given the critical role of CTSC in HCC radiotherapy resistance, we investigated whether inhibiting the CTSC signalling pathway could enhance HCC radiosensitivity. AZD7986, a second generation CTSC inhibitor, has shown promise in treating liver injury and tumours [14, 31]. In the experiment, AZD7986 (5 mg/kg) was administered orally twice daily until the mice were sacrificed [32]. The combination of AZD7986 and radiotherapy significantly inhibited tumour growth compared to radiotherapy alone (Figure 6A). AZD7986 had no significant effect on the body weight of mice and the morphology of the liver and kidneys in HE staining, indicating that the drug has good tolerability (Figure 6B,C). To further analyse the underlying immune mechanisms, we used IHC to assess immune cell infiltration in tumour tissues. IHC analysis revealed that the combination therapy significantly reduced MDSC infiltration while increasing CD8^+^ T cell numbers (Figure 6C). Additionally, in the orthotopic liver tumour model, the combination of AZD7986 and radiotherapy significantly reduced tumour size in Hepa1‐6‐CTSC cells compared to radiotherapy alone (Figure 6D,E). These results suggest that pharmacological inhibition of CTSC enhances radiotherapy efficacy, potentially through adaptive immunity.

## 4 Discussion

Advancements in radiotherapy technology have increasingly positioned radiotherapy as a pivotal treatment modality for unresectable or advanced HCC. However, radioresistance remains a significant challenge, limiting its efficacy in HCC patients [5, 33]. Previous studies have established that MDSC infiltration is closely associated with tumour recurrence, proliferation and progression. In this study, we demonstrated that CTSC is highly expressed in HCC and is strongly correlated with high recurrence rates, high metastasis rates and poor overall survival. Furthermore, in vitro and in vivo experiments confirmed that CTSC reduces HCC radiosensitivity by recruiting MDSCs, highlighting its critical role in mediating radiotherapy resistance in HCC.

Radiotherapy exerts complex effects on the tumour microenvironment: while it can trigger DNA and RNA sensing cascades to activate innate immunity and enhance adaptive immune responses, it may also suppress immune‐active cells and recruit immunosuppressive populations, leading to immune evasion [18, 34]. Both preclinical and clinical studies have shown that MDSCs are closely associated with disease progression and radiotherapy resistance in cancer patients. Therefore, inhibiting MDSCs is a promising strategy to enhance the efficacy of radiotherapy and immunotherapy [8]. However, the role of MDSCs in radiotherapy resistance, particularly in HCC, remains poorly understood. Our findings revealed that CTSC promotes radiotherapy resistance in HCC by upregulating CXCL1 expression and recruiting MDSCs. These results underscore the critical role of MDSCs in HCC radiotherapy resistance.

In recent years, the combination of radiotherapy with other therapies has garnered significant attention in HCC treatment [2, 35]. Radiotherapy can enhance antigen presentation and tumour immunogenicity, thereby modulating tumour phenotypes and improving the efficacy of immunotherapy. Increasingly, studies have explored the combination of radiotherapy and immunotherapy to achieve better treatment outcomes. In this study, we found that combining the CTSC inhibitor AZD7986 with radiotherapy significantly improved therapeutic efficacy in both subcutaneous and orthotopic liver tumour models. IHC analysis showed that the combination therapy reduced MDSC infiltration and increased CD8^+^ T cell infiltration, suggesting that the antitumor effects of CTSC inhibition may rely on adaptive immunity. Regretfully, the relationship between the expression of CTSC and the efficacy of radiotherapy was investigated with only 30 samples, and further validation is required in subsequent studies. In addition, our in vitro clonogenic survival results suggest that CTSC may also exert a cell intrinsic effect on HCC cells to enhance radiation resistance, independent of immune cell infiltration, which will be further investigated in the following study.

In conclusion, we have identified a novel function of CTSC in promoting radiotherapy resistance in HCC. CTSC overexpression recruits MDSCs by upregulating CXCL1, thereby promoting HCC metastasis and radiotherapy resistance. Combining CTSC inhibition with radiotherapy significantly enhances HCC radiotherapy efficacy. Our findings suggest that CTSC could serve as a potential prognostic biomarker for HCC, and targeting this signalling pathway offers a promising therapeutic approach for CTSC‐mediated radiotherapy resistance in HCC.

## Author Contributions

**Jiahuan Xu:** data curation, investigation. **Jie Wu:** formal analysis, supervision. **Bilin Zhang:** methodology. **Shirui Yang:** methodology, visualization. **Yunzhi Dang:** conceptualization, writing – original draft, writing – review and editing, project administration. **Shaoran Song:** data curation, investigation, supervision.

## Funding

This work was supported by Fundamental Research Funds for the Central Universities, xzy012025137. National Natural Science Foundation of China, 82203521, 82503613. Incubation Fund Program of Shaanxi Provincial People's Hospital, 2023YJY‐03. Social Development Field in General Project of Key Research and Development Program of Shaanxi Province, 2025SF‐YBXM‐347, 2025SF‐YBXM‐395.

## Ethics Statement

This study was approved by the Ethics Committee of Shaanxi Provincial People's Hospital (approval number, 2025R064). All procedures were conducted in accordance with the ethical guidelines of the 1975 Declaration of Helsinki.

## Consent

The authors have nothing to report.

## Conflicts of Interest

The authors declare no conflicts of interest.

## Supporting information


**Figure S1:** CTSC cannot promote HCC metastasis in nude mice. (A). Transwell assay analysis of the invasion and metastasis abilities of Huh7‐control cells and Huh7‐CTSC cells. (B, C). In vivo metastasis assays in nude mice. (B). Bioluminescent images. (C). Representative HE staining.
**Table S1:** List of genes differentially expressed in Huh7‐CTSC versus Huh7‐control cells using a Affymetrix PrimeView Human gene expression array.

## Data Availability

The data that support the findings of this study are available on request from the corresponding author. The data are not publicly available due to privacy or ethical restrictions.
