# Supplementary material for: CTSC Confers Radioresistance in Hepatocellular Carcinoma by Regulating Myeloid‐Derived Suppressor Cells
Source: J Cell Mol Med. 2026 Apr 21;30(8):e71153. doi: 10.1111/jcmm.71153 (PMC13099595; doi:10.1111/jcmm.71153)
Supplement: Supplementary file 1 — Figure S1: CTSC cannot promote HCC metastasis in nude mice. (A). Transwell assay analysis of the invasion and metastasis abilities of Huh7‐control cells and Huh7‐CTSC cells. (B, C). In vivo metastasis assays in nude mice. (B). Bioluminescent images. (C). Representative HE staining. Table S1: List of genes differentially expressed in Huh7‐CTSC versus Huh7‐control cells using a Affymetrix PrimeView Human gene expression array. [file JCMM-30-e71153-s001.docx]

**CTSC Confers Radioresistance in Hepatocellular Carcinoma by Regulating Myeloid-derived Suppressor Cells**

Jiahuan Xu^1,2^†, Bilin Zhang^1,2^†, Shirui Yang^1,3^†, Shaoran Song^1^, Jie Wu^1^, Yunzhi Dang^1,4✉^

1. Department of Radiation Oncology, Shaanxi Provincial People’s Hospital, Xi’an, Shaanxi, 710086, China

2. Xi’an Medical University, Xi’an, Shaanxi, 710086, China

3. Medical School of Yan’an University, Yan’an, Shaanxi, 716000, China

4. State Key Laboratory of Cancer Biology, Fourth Military Medical University, Xi’an 710032, China

†These authors contributed equally to this work.

**Corresponding author**:

Yunzhi Dang, Department of Radiation Oncology, Shaanxi Provincial People’s Hospital, Xi’an 710086, China.

**E-mail**: dangyunzhi@xiyi.edu.cn

**Supplementary Material and Methods**

**Cells and culture**

In this research, the human hepatocellular carcinoma cell lines Huh7 and HCCLM3 were procured from the American Type Culture Collection (ATCC). The Hepa1-6 cells, which originated from the BW7756 tumor of C57BL/C mice, were also sourced from ATCC (Manassas, VA, USA).The Hepa1-6 cells were maintained in Dulbecco’s Modified Eagle Medium (DMEM, Gibco). The culture medium was fortified with 10% fetal bovine serum (FBS, Gibco), 100 μg/ml penicillin, and 100 μg/ml streptomycin [27].

**Construction of lentivirus and stable cell lines**

Construction of lentivirus and stable cell lines Lentiviral vectors encoding shRNAs were generated using PLKO.1-TRC (Addgene) and designated as LV-shCTSC (mice), LV-shCTSC (human), and LV-shcontrol. “LV-shcontrol” is a non-target shRNA control. The vector “pLKO.1-puro non-Target shRNA Control Plasmid DNA” (purchased from Sigma, SHC016) contains an shRNA insert that does not target any known genes from any species. Short hairpin RNAs (shRNAs) sequences were: shCTSC (human), 5’-GCTGCTACTCATTTGCTTCTA-3’.

Lentiviral vectors encoding the mice and human CTSC genes were constructed in FUW-teto (Addgene) and designated as LV-CTSC. An empty vector was used as the negative control and was designated as LV-control. Concentrated lentivirus was transfected into the HCC cells with a multiplicity of infection (MOI) ranging from 30 to 50 in the presence of polybrene (6 μg/ml). Seventy-two hours after infection, HCC cells were selected for 2 weeks using 2.5 μg/ml puromycin (OriGene). Selected pools of knockdown and overexpression cells were used for the follow experiments.

**Real-time PCR**

Total RNA was extracted using TRIzol Reagent (Invitrogen), and reverse transcription was performed using the Advantage for RT-PCR Kit (Takara) according to the manufacturer’s instructions. For the real-time PCR analysis, aliquots of double-stranded cDNA were amplified using a SYBR Green PCR Kit (Applied Biosystems). For the clinical tissue samples, the fold change of the target gene was determined by the following equation: 2^–ΔΔCt^ (ΔΔCt=ΔCt^tumor^–ΔCt^nontumor^). This value was normalized to the average fold change in the normal liver tissues, which was defined as 1.0. All reactions were performed in duplicate. The primer sequences for CTSC sense were 5’-CCAACTGCACCTATCTTGACC-3’, CTSC antisense was 5’-AAGGCAAACCACTTGTAGTCATT-3’.

**In vivo metastatic model and bioluminescent imaging**

Luciferase lentivirus was purchased from Shanghai Genechem Co, Ltd. Concentrated luciferase lentivirus was transfected into the HCC cells with a multiplicity of infection (MOI=50) in the presence of polybrene (6 μg/ml). Seventy-two hours after infection, HCC cells were selected for 2 weeks using 2.5 μg/ml puromycin (OriGene). Then we tested the luciferase infection efficiency. In a 96-well plate, we set up 4 gradient dilution cells (each hole is spaced at a certain distance to prevent mutual interference). Then, 5 μl D-luciferin was added to each hole, and the signal value of each well was measured by a multifunctional enzyme marker. If the cell density were positively correlated with the signal value, indicated luciferase transfection success.

The in vivo tumor formation and metastases were imaged by bioluminescence. D-luciferin (Xenogen, Hopkinton, MA) at 100 mg/kg was injected intraperitoneally into the mice, and bioluminescence was detected using an IVIS 100 Imaging System (Xenogen). After acquiring photographic images of each mouse, luminescent photos were captured using various (1-60 seconds) exposure times. The resulting grayscale photographic and pseudocolored luminescent images were automatically superimposed using the IVIS Living Image (Xenogen) software. This superimposition was performed to facilitate the matching of the observed luciferase signal with its location on the mouse.

**Preparation of Single Cell Suspensions**

Mice were perfused with PBS and anesthetized, and tumors were dissected using a clean razor. Then, the tumor tissues were digested with DNase I (20 mg/mL, Sigma-Aldrich) and collagenase IV (1.5 mg/mL, Sigma-Aldrich) and placed on a table concentrator, 37℃, for one hour. At the end of one hour, we filtered the dissociated cells through 70 μm pore filters rinsed with fresh media. The 1×red cell lysis was added to the tissues and incubated for 5 minutes to lysis the red blood cell, followed by another rinse.

**Flow Cytometric Analysis**

Cells were incubated with anti-mouse CD16/CD32 purified antibody (#101302, clone 93, Biolegend) for 10 minutes to block nonspecific antibodies. Then, the cells were stained with fluorophore-conjugated antibodies. Matched isotype antibodies were used as control. Antibodies against CD45 (PE, #103105), CD11b (FITC, #101205), CD45 (PE/Cy7, #103113), Ly-6G/Ly-6C (Gr-1) (PE, #108407), CD3 (FITC, #100203), CD8 (PE, #100707), F4/80 (PE/Cy7, #123113), were purchased from biolegend. Data were analyzed by Flowjo_V10 software (TreeStar, Ashland, OR).

**Supplementary Figure and Table**


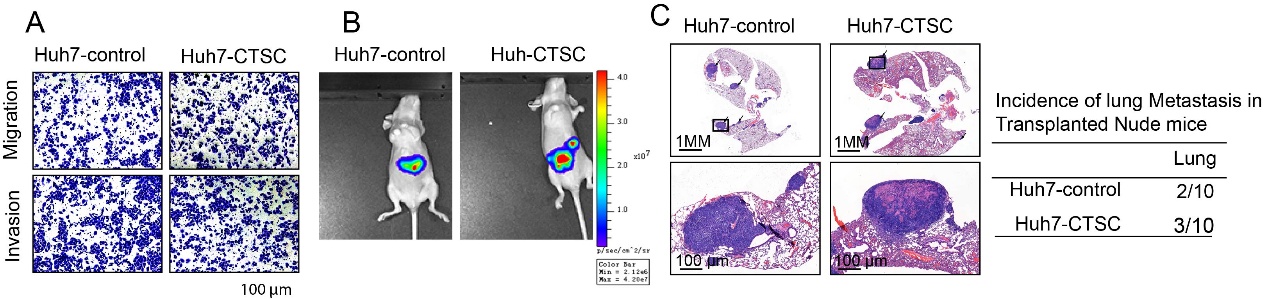


Supplementary Figure 1. CTSC cannot promote HCC metastasis in nude mice.

(A). Transwell assay analysis of the invasion and metastasis abilities of Huh7-control cells and Huh7-CTSC cells.

(B-C). In vivo metastasis assays in nude mice. (B). Bioluminescent images. (C). Representative HE staining.

**Supplementary table 1. List of genes differentially expressed in Huh7-CTSC versus Huh7-control cells using a** **Affymetrix PrimeView Human gene expression array**

| **Gene Symbol** | **Description** | **Fold Change** |
| --- | --- | --- |
| ASB4 | ankyrin repeat and SOCS box containing 4 | 3.95 |
| HIST1H2BJ | histone cluster 1, H2bj | 3.84 |
| EHF | ets homologous factor | 3.55 |
| EHF | ets homologous factor | 3.53 |
| CD163L1 | CD163 molecule-like 1 | 3.49 |
| GDA | guanine deaminase | 3.36 |
| HBE1 | hemoglobin, epsilon 1 | 3.3 |
| CXCL1 | chemokine (C-X-C motif) ligand 1 | 3.07 |
| SPANXB1 | SPANX family, member B1 | 3.04 |
| TKTL1 | transketolase-like 1 | 2.92 |
| TNNC1 | troponin C type 1 (slow) | 2.88 |
| RPUSD4 | RNA pseudouridylate synthase domain containing 4 | 2.84 |
| HIST1H1C | histone cluster 1, H1c | 2.82 |
| TFRC | transferrin receptor | 2.8 |
| ICAM1 | Intercellular Adhesion Molecule1 | 2.76 |
| HIST1H2BJ | histone cluster 1, H2bj | 2.72 |
| SLC16A14 | solute carrier family 16, member 14 | 2.71 |
| CTPS1 | CTP synthase 1 | 2.69 |
| EIF4A2 | eukaryotic translation initiation factor 4A2 | 2.69 |
| NFIA | nuclear factor I/A | 2.66 |
| RPUSD4 | RNA pseudouridylate synthase domain containing 4 | 2.62 |
| UHRF1 | ubiquitin-like with PHD and ring finger domains 1 | 2.61 |
| RPSA | ribosomal protein SA | 2.61 |
| IER3IP1 | immediate early response 3 interacting protein 1 | 2.58 |
| NAA15 | N(alpha)-acetyltransferase 15, NatA auxiliary subunit | 2.57 |
| XK | X-linked Kx blood group | 2.55 |
| NAA15 | N(alpha)-acetyltransferase 15 | 2.54 |
| RAN | RAN, member RAS oncogene family | 2.54 |
| ASB4 | ankyrin repeat and SOCS box containing 4 | 2.52 |
| RCAN1 | regulator of calcineurin 1 | 2.5 |
| MZT1 | mitotic spindle organizing protein 1 | 2.49 |
| MCM6 | minichromosome maintenance complex component 6 | 2.48 |
| RPP40 | ribonuclease P/MRP 40kDa subunit | 2.47 |
| TMEM110 | transmembrane protein 110 | 2.47 |
| RFWD3 | ring finger and WD repeat domain 3 | 2.47 |
| GANAB | glucosidase, alpha; neutral AB | 2.46 |
| RPL36 | ribosomal protein L36 | 2.45 |
| GRAMD3 | GRAM domain containing 3 | 2.45 |
| KBTBD8 | kelch repeat and BTB (POZ) domain containing 8 | 2.43 |
| CTPS1 | CTP synthase 1 | 2.42 |
| TNFRSF21 | tumor necrosis factor receptor superfamily, member 21 | 2.42 |
| PDE12 | phosphodiesterase 12 | 2.4 |
| PRR20 | proline rich 20 | 2.39 |
| MAPK9 | mitogen-activated protein kinase 9 | 2.39 |
| HSPA8 | heat shock 70kDa protein 8 | 2.39 |
| GPATCH4 | G-patch domain containing 4 | 2.38 |
| MZT1 | mitotic spindle organizing protein 1 | 2.38 |
| FAM169A | family with sequence similarity 169, member A | 2.38 |
| KCTD20 | potassium channel tetramerization domain containing 20 | 2.38 |
| LANCL1 | LanC lantibiotic synthetase component C-like 1 | 2.37 |
| SCARNA14 | small Cajal body-specific RNA 14 | 2.36 |
| CD24 | CD24 molecule | 2.35 |
| CTPS1 | CTP synthase 1 | 2.35 |
| TENM2 | teneurin transmembrane protein 2 | 2.34 |
| SRM | spermidine synthase | 2.33 |
| CXCR2 | C-X-C chemokine receptor type 2 | 2.33 |
| SERP1 | stress-associated endoplasmic reticulum protein 1 | 2.31 |
| RPL27A | ribosomal protein L27a | 2.31 |
| POLR3G | polymerase (RNA) III (DNA directed) polypeptide G | 2.31 |
| RTN4IP1 | reticulon 4 interacting protein 1 | 2.3 |
| APBB2 | amyloid beta (A4) precursor protein-binding, family B, member 2 | 2.3 |
| SMIM10 | small integral membrane protein 10 | 2.3 |
| PDE12 | phosphodiesterase 12 | 2.29 |
| MCM10 | minichromosome maintenance 10 replication initiation factor | 2.28 |
| CTPS1 | CTP synthase 1 | 2.28 |
| SLC7A11 | Solute Carrier Family 7 Member 11 | 2.27 |
| GINS2 | GINS complex subunit 2 (Psf2 homolog) | 2.27 |
| GPAM | glycerol-3-phosphate acyltransferase, mitochondrial | 2.26 |
| NCS1 | neuronal calcium sensor 1 | 2.25 |
| TMEM201 | transmembrane protein 201 | 2.25 |
| POLR3H | polymerase (RNA) III (DNA directed) polypeptide H | 2.24 |
| SERP1 | stress-associated endoplasmic reticulum protein 1 | 2.24 |
| SERP1 | stress-associated endoplasmic reticulum protein 1 | 2.24 |
| POLR3H | polymerase (RNA) III (DNA directed) polypeptide H | 2.24 |
| HSPA8 | heat shock 70kDa protein 8 | 2.24 |
| PNPO | pyridoxamine 5'-phosphate oxidase | 2.23 |
| ANAPC7 | anaphase promoting complex subunit 7 | 2.22 |
| PHLDB2 | pleckstrin homology-like domain, family B, member 2 | 2.22 |
| UHRF1 | ubiquitin-like with PHD and ring finger domains 1 | 2.21 |
| NEK3 | NIMA-related kinase 3 | 2.21 |
| UTP15 | UTP15, U3 small nucleolar ribonucleoprotein, homolog | 2.2 |
| SNHG15; SNORA9 | small nucleolar RNA host gene 15; small nucleolar RNA, H/ACA box 9 | 2.2 |
| RBP1 | retinol binding protein 1, cellular | 2.2 |
| SCFD2 | sec1 family domain containing 2 | 2.19 |
| PDE3B | phosphodiesterase 3B, cGMP-inhibited | 2.19 |
| SNRNP25 | small nuclear ribonucleoprotein | 2.18 |
| SIX1 | SIX homeobox 1 | 2.18 |
| LETM1 | leucine zipper-EF-hand containing transmembrane protein 1 | 2.17 |
| C18orf25 | chromosome 18 open reading frame 25 | 2.17 |
| SGPP2 | sphingosine-1-phosphate phosphatase 2 | 2.17 |
| RBP1 | retinol binding protein 1, cellular | 2.17 |
| UGT1A | UDP glucuronosyltransferase 1 family, polypeptide A | 2.17 |
| LRP11 | LDL receptor related protein 11 | 2.17 |
| RAB31 | RAB31, member RAS oncogene family | 2.16 |
| RWDD4 | RWD domain containing 4 | 2.16 |
| NACAP1 | nascent-polypeptide-associated complex alpha polypeptide pseudogene 1 | 2.16 |
| ELOVL6 | ELOVL fatty acid elongase 6 | 2.15 |
| ZMYND11 | zinc finger, MYND-type containing 11 | 2.15 |
| RPS14 | ribosomal protein S14 | 2.14 |
| RPUSD4 | RNA pseudouridylate synthase domain containing 4 | 2.14 |
| CHORDC1 | cysteine and histidine rich domain containing 1 | 2.14 |
| CFL1 | cofilin 1 (non-muscle) | 2.14 |
| UCHL5 | ubiquitin C-terminal hydrolase L5 | 2.14 |
| SKP2 | S-phase kinase-associated protein 2, E3 ubiquitin protein ligase | 2.13 |
| FAM169A | family with sequence similarity 169, member A | 2.12 |
| ELAC1 | elaC ribonuclease Z 1 | 2.11 |
| NSG1 | neuron specific gene family member 1 | 2.11 |
| PHLDB2 | pleckstrin homology-like domain, family B, member 2 | 2.11 |
| OTUD6B | OTU domain containing 6B | 2.11 |
| DEF8 | differentially expressed in FDCP 8 homolog | 2.1 |
| BTF3L4 | basic transcription factor 3-like 4 | 2.1 |
| TIPIN | TIMELESS interacting protein | 2.1 |
| ONECUT2 | one cut homeobox 2 | 2.1 |
| TAF9B | TAF9B RNA polymerase II | 2.1 |
| ELOVL6 | ELOVL fatty acid elongase 6 | 2.1 |
| SNORA13 | small nucleolar RNA, H/ACA box 13 | 2.1 |
| SERP1 | stress-associated endoplasmic reticulum protein 1 | 2.09 |
| CERS6 | ceramide synthase 6 | 2.09 |
| HIST2H4A; | histone cluster 2, H4a | 2.09 |
| HIST2H4B | histone cluster 2, H4b | 2.09 |
| DENND1A | DENN/MADD domain containing 1A | 2.09 |
| EIF4A2 | eukaryotic translation initiation factor 4A2 | 2.09 |
| DTL | denticleless E3 ubiquitin protein ligase homolog | 2.09 |
| CSNK2A2 | casein kinase 2, alpha prime polypeptide | 2.09 |
| FABP5 | fatty acid binding protein 5 | 2.08 |
| DEF8 | differentially expressed in FDCP 8 homolog | 2.08 |
| CST1 | cystatin SN | 2.08 |
| FKBP4 | FK506 binding protein 4 | 2.08 |
| B3GALNT1 | beta-1,3-N-acetylgalactosaminyltransferase 1 | 2.08 |
| HAUS7 | HAUS augmin like complex subunit 7 | 2.08 |
| SLC39A9 | solute carrier family 39, member 9 | 2.08 |
| LTV1 | LTV1 ribosome biogenesis factor | 2.08 |
| CFL1 | cofilin 1 (non-muscle) | 2.08 |
| ARL4A | ADP-ribosylation factor like GTPase 4A | 2.07 |
| CHORDC1 | cysteine and histidine rich domain containing 1 | 2.07 |
| ZC3HAV1 | zinc finger CCCH-type, antiviral 1 | 2.07 |
| PDSS1 | prenyl (decaprenyl) diphosphate synthase, subunit 1 | 2.06 |
| CREM | cAMP responsive element modulator | 2.06 |
| TRIP13 | thyroid hormone receptor interactor 13 | 2.06 |
| RASL11B | RAS-like, family 11, member B | 2.06 |
| EP400NL | EP400 N-terminal like | 2.06 |
| RDH10 | retinol dehydrogenase 10 (all-trans) | 2.05 |
| TIGAR | TP53 induced glycolysis regulatory phosphatase | 2.05 |
| SIX1 | SIX homeobox 1 | 2.05 |
| C9orf40 | chromosome 9 open reading frame 40 | 2.05 |
| SLC25A15 | solute carrier family 25 member 15 | 2.05 |
| TKTL1 | transketolase-like 1 | 2.05 |
| HNRNPC | heterogeneous nuclear ribonucleoprotein C (C1/C2) | 2.05 |
| HIST1H2BK | histone cluster 1, H2bk | 2.04 |
| CXCL14 | chemokine (C-X-C motif) ligand 14 | 2.04 |
| FABP5 | fatty acid binding protein 5 | 2.04 |
| AADAT | aminoadipate aminotransferase | 2.04 |
| MFAP3L | microfibrillar associated protein 3 like | 2.04 |
| MCM6 | minichromosome maintenance complex component 6 | 2.04 |
| CFL1 | cofilin 1 (non-muscle) | 2.04 |
| EIF4A2 | eukaryotic translation initiation factor 4A2 | 2.04 |
| RAB31 | RAB31, member RAS oncogene family | 2.04 |
| RPS14 | ribosomal protein S14 | 2.03 |
| CEP83 | centrosomal protein | 2.03 |
| RCC1 | regulator of chromosome condensation 1 | 2.03 |
| ERVMER34-1 | endogenous retrovirus group MER34, member 1 | 2.03 |
| CCDC59 | coiled-coil domain containing 59 | 2.03 |
| POLR3H | polymerase (RNA) III (DNA directed) polypeptide H | 2.02 |
| MGST1 | microsomal glutathione S-transferase 1 | 2.02 |
| TRIP13 | thyroid hormone receptor interactor 13 | 2.02 |
| DZIP3 | DAZ interacting zinc finger protein 3 | 2.02 |
| PDE12 | phosphodiesterase 12 | 2.02 |
| PTPN2 | protein tyrosine phosphatase, non-receptor type 2 | 2.02 |
| SETD7 | SET domain containing (lysine methyltransferase) 7 | 2.01 |
| CASP2 | caspase 2 | 2.01 |
| LRRC59 | leucine rich repeat containing 59 | 2.01 |
| F3 | coagulation factor III (thromboplastin, tissue factor) | 2.01 |
| GNAI1 | guanine nucleotide binding protein (G protein), alpha inhibiting activity polypeptide 1 | 2.01 |
| STRA6 | stimulated by retinoic acid 6 | 2.01 |
| GANAB | glucosidase, alpha; neutral AB | 2.01 |
| COPS8 | COP9 signalosome subunit 8 | 2.01 |
| ATAD3A; | ATPase family, AAA domain containing 3A | 2 |
| ATAD3B | ATPase family, AAA domain containing 3B | 2 |
| PNPO | pyridoxamine 5'-phosphate oxidase | 2 |
| UGT1A | UDP glucuronosyltransferase 1 family, polypeptide A | 2 |
| FAM229B | family with sequence similarity 229, member B | 2 |
| LMNB1 | lamin B1 | 2 |
| C1orf112 | chromosome 1 open reading frame 112 | 2 |
| CYP2U1 | cytochrome P450, family 2, subfamily U, polypeptide 1 | 2 |
| CREBRF | CREB3 regulatory factor | -2 |
| GSN | gelsolin | -2 |
| TDRD6 | tudor domain containing 6 | -2 |
| PKD1L2 | polycystic kidney disease 1-like 2 | -2 |
| MEGF6 | multiple EGF-like-domains 6 | -2.01 |
| PROC | protein C (inactivator of coagulation factors Va and VIIIa) | -2.01 |
| ALDH1A3 | aldehyde dehydrogenase 1 family, member A3 | -2.01 |
| VEGFA | vascular endothelial growth factor A | -2.02 |
| BACH1; GRIK1-AS2 | BTB and CNC homology 1, basic leucine zipper transcription factor 1; GRIK1 antisense RNA 2 | -2.02 |
| BNIP3L | BCL2/adenovirus E1B 19kDa interacting protein 3-like | -2.02 |
| YPEL4 | yippee like 4 | -2.02 |
| UCN | urocortin | -2.03 |
| SDK1 | sidekick cell adhesion molecule 1 | -2.03 |
| RNF24 | ring finger protein 24 | -2.03 |
| ALDOC | aldolase C, fructose-bisphosphate | -2.03 |
| ALDH1A3 | aldehyde dehydrogenase 1 family, member A3 | -2.03 |
| ALDOC | aldolase C, fructose-bisphosphate | -2.03 |
| CHST15 | carbohydrate sulfotransferase 15 | -2.03 |
| CADM1 | cell adhesion molecule 1 | -2.03 |
| STC2 | stanniocalcin 2 | -2.04 |
| KIT | v-kit Hardy-Zuckerman 4 feline sarcoma viral oncogene homolog | -2.04 |
| PAEP | progestagen-associated endometrial protein | -2.04 |
| FGF11 | fibroblast growth factor 11 | -2.04 |
| FN1 | fibronectin 1 | -2.04 |
| PARD3 | par-3 family cell polarity regulator | -2.04 |
| ZNF395 | zinc finger protein 395 | -2.05 |
| KIAA1217 | KIAA1217 | -2.05 |
| DSC2 | desmocollin 2 | -2.05 |
| ALDH1A3 | aldehyde dehydrogenase 1 family, member A3 | -2.05 |
| IL11RA | interleukin 11 receptor, alpha | -2.06 |
| FAM129A | family with sequence similarity 129, member A | -2.06 |
| FN1 | fibronectin 1 | -2.06 |
| BNIP3L | BCL2/adenovirus E1B 19kDa interacting protein 3-like | -2.07 |
| IGF1R | insulin-like growth factor 1 receptor | -2.07 |
| NT5E | 5'-nucleotidase, ecto (CD73) | -2.07 |
| SERPINA1 | serpin peptidase inhibitor, clade A (alpha-1 antiproteinase, antitrypsin), member 1 | -2.07 |
| PPARA | peroxisome proliferator-activated receptor alpha | -2.07 |
| FAM107B | family with sequence similarity 107, member B | -2.07 |
| EHD2 | EH domain containing 2 | -2.07 |
| SYNJ2 | synaptojanin 2 | -2.07 |
| C4orf47 | chromosome 4 open reading frame 47 | -2.07 |
| CADM1 | cell adhesion molecule 1 | -2.07 |
| MT1F | metallothionein 1F | -2.08 |
| LAPTM5 | lysosomal protein transmembrane 5 | -2.08 |
| TNFAIP8 | tumor necrosis factor, alpha-induced protein 8 | -2.08 |
| KRT17 | keratin 17, type I | -2.09 |
| YPEL3 | yippee like 3 | -2.09 |
| KRT15 | keratin 15, type I | -2.09 |
| PNRC1 | proline-rich nuclear receptor coactivator 1 | -2.09 |
| CREBRF | CREB3 regulatory factor | -2.09 |
| TSC22D3 | TSC22 domain family, member 3 | -2.1 |
| GAL3ST1 | galactose-3-O-sulfotransferase 1 | -2.1 |
| CCNG2 | cyclin G2 | -2.1 |
| ELF3 | E74-like factor 3 | -2.1 |
| WDR26 | WD repeat domain 26 | -2.1 |
| YPEL5 | yippee like 5 | -2.11 |
| FRMD4A | FERM domain containing 4A | -2.11 |
| VEGFA | vascular endothelial growth factor A | -2.11 |
| FAM183A | family with sequence similarity 183, member A | -2.11 |
| INHBE | inhibin beta E | -2.11 |
| BNIP3L | BCL2/adenovirus E1B 19kDa interacting protein 3-like | -2.11 |
| BNIP3L | BCL2/adenovirus E1B 19kDa interacting protein 3-like | -2.11 |
| SLC2A14 | solute carrier family 2 (facilitated glucose transporter), member 14 | -2.11 |
| CSRNP1 | cysteine-serine-rich nuclear protein 1 | -2.12 |
| PIK3IP1 | phosphoinositide-3-kinase interacting protein 1 | -2.12 |
| DUSP5 | dual specificity phosphatase 5 | -2.13 |
| PDZD2 | PDZ domain containing 2 | -2.13 |
| INHA | inhibin alpha | -2.13 |
| CCNG2 | cyclin G2 | -2.13 |
| PARD3 | par-3 family cell polarity regulator | -2.13 |
| N4BP2L1 | NEDD4 binding protein 2-like 1 | -2.13 |
| BNIP3L | BCL2/adenovirus E1B 19kDa interacting protein 3-like | -2.13 |
| CCL28 | chemokine (C-C motif) ligand 28 | -2.13 |
| KCNN4 | potassium channel, calcium activated intermediate/small conductance subfamily N alpha, member 4 | -2.13 |
| KLF9 | Kruppel-like factor 9 | -2.14 |
| PLA2G16 | phospholipase A2, group XVI | -2.14 |
| NAV1 | neuron navigator 1 | -2.14 |
| PADI1 | peptidyl arginine deiminase, type I | -2.14 |
| WFDC3 | WAP four-disulfide core domain 3 | -2.14 |
| SLC5A1 | solute carrier family 5 (sodium/glucose cotransporter), member 1 | -2.14 |
| CADM1 | cell adhesion molecule 1 | -2.14 |
| MIG7 | mig-7 | -2.14 |
| COL6A1 | collagen, type VI, alpha 1 | -2.15 |
| PDZD2 | PDZ domain containing 2 | -2.15 |
| ANKH | ANKH inorganic pyrophosphate transport regulator | -2.15 |
| INSR | insulin receptor | -2.15 |
| SERPINA1 | serpin peptidase inhibitor, clade A (alpha-1 antiproteinase, antitrypsin), member 1 | -2.15 |
| UBASH3B | ubiquitin associated and SH3 domain containing B | -2.15 |
| STON1 | stonin 1 | -2.15 |
| KIAA0040 | KIAA0040 | -2.15 |
| EMP1 | epithelial membrane protein 1 | -2.16 |
| PCMTD1 | protein-L-isoaspartate (D-aspartate) O-methyltransferase domain containing 1 | -2.16 |
| FBXO16 | F-box protein 16; zinc finger protein 395 | -2.16 |
| LBH | limb bud and heart development | -2.17 |
| CEMIP | cell migration inducing protein, hyaluronan binding | -2.17 |
| TNFRSF9 | tumor necrosis factor receptor superfamily, member 9 | -2.17 |
| ZBTB20 | zinc finger and BTB domain containing 20 | -2.17 |
| INSIG2 | insulin induced gene 2 | -2.18 |
| MT1F | metallothionein 1F | -2.18 |
| TRPV4 | transient receptor potential cation channel, subfamily V, member 4 | -2.18 |
| CD300A | CD300a molecule | -2.18 |
| SMAD6 | SMAD family member 6 | -2.18 |
| HYAL1 | hyaluronoglucosaminidase 1 | -2.18 |
| TRPV4 | transient receptor potential cation channel, subfamily V, member 4 | -2.18 |
| PARD3 | par-3 family cell polarity regulator | -2.18 |
| EMP1 | epithelial membrane protein 1 | -2.19 |
| SLC2A14; | solute carrier family 2 (facilitated glucose transporter), member 14 | -2.19 |
| SLC2A3 | solute carrier family 2 (facilitated glucose transporter), member 3 |  |
| COL13A1 | collagen, type XIII, alpha 1 | -2.19 |
| FER1L4 | fer-1-like family member 4, pseudogene | -2.19 |
| C20orf195 | chromosome 20 open reading frame 195 | -2.2 |
| IGF1R | insulin-like growth factor 1 receptor | -2.2 |
| GBE1 | glucan (1,4-alpha-), branching enzyme 1 | -2.21 |
| TRIM9 | tripartite motif containing 9 | -2.21 |
| FAM13A | family with sequence similarity 13, member A | -2.21 |
| ABCC3 | ATP binding cassette subfamily C member 3 | -2.21 |
| ALDH1A3 | aldehyde dehydrogenase 1 family, member A3 | -2.21 |
| BEST4 | bestrophin 4 | -2.22 |
| CASP4 | caspase 4 | -2.22 |
| INSIG2 | insulin induced gene 2 | -2.23 |
| ARG2 | arginase 2 | -2.23 |
| WFDC3 | WAP four-disulfide core domain 3 | -2.23 |
| ARG2 | arginase 2 | -2.23 |
| FOSL2 | FOS-like antigen 2 | -2.24 |
| NPNT | nephronectin | -2.25 |
| INSIG2 | insulin induced gene 2 | -2.25 |
| SERPINA1 | serpin peptidase inhibitor, clade A (alpha-1 antiproteinase, antitrypsin), member 1 | -2.25 |
| FRMD4A | FERM domain containing 4A | -2.25 |
| FUT11 | fucosyltransferase 11 | -2.25 |
| LIMCH1 | LIM and calponin homology domains 1 | -2.25 |
| SPARC | secreted protein, acidic, cysteine-rich (osteonectin) | -2.26 |
| ANG | angiogenin, ribonuclease, RNase A family, 5 | -2.26 |
| ITGA5 | integrin alpha 5 | -2.27 |
| YPEL3 | yippee like 3 | -2.28 |
| CREBRF | CREB3 regulatory factor | -2.28 |
| CREBRF | CREB3 regulatory factor | -2.28 |
| HBP1 | HMG-box transcription factor 1 | -2.29 |
| EGLN3 | egl-9 family hypoxia-inducible factor 3 | -2.29 |
| KIAA0040 | KIAA0040 | -2.29 |
| S100A3 | S100 calcium binding protein A3 | -2.3 |
| SLC16A4 | solute carrier family 16, member 4 | -2.3 |
| KCNJ10 | potassium channel, inwardly rectifying subfamily J, member 10 | -2.3 |
| STC2 | stanniocalcin 2 | -2.31 |
| LPCAT2 | lysophosphatidylcholine acyltransferase 2 | -2.31 |
| PLOD2 | Procollagen lysine, 2-oxoglutarate 5-dioxygenase 2 | -2.32 |
| COL6A1 | collagen, type VI, alpha 1 | -2.33 |
| SEC14L2 | SEC14-like lipid binding 2 | -2.33 |
| CLEC2D | C-type lectin domain family 2, member D | -2.33 |
| TRIOBP | TRIO and F-actin binding protein | -2.34 |
| SYTL3 | synaptotagmin-like 3 | -2.34 |
| CCNG2 | cyclin G2 | -2.35 |
| SMIM5 | small integral membrane protein 5 | -2.35 |
| SMIM5 | small integral membrane protein 5 | -2.35 |
| COL6A1 | collagen, type VI, alpha 1 | -2.37 |
| SLC16A6 | solute carrier family 16, member 6 | -2.38 |
| KLHL24 | kelch-like family member 24 | -2.38 |
| IGF1R | insulin-like growth factor 1 receptor | -2.39 |
| SPAG4 | sperm associated antigen 4 | -2.39 |
| CCL26 | chemokine (C-C motif) ligand 26 | -2.4 |
| CYP3A5 | cytochrome P450, family 3, subfamily A, polypeptide 5 | -2.4 |
| AHNAK2 | AHNAK nucleoprotein 2 | -2.4 |
| ISG20 | interferon stimulated exonuclease gene 20kDa | -2.4 |
| BHLHE41 | basic helix-loop-helix family, member e41 | -2.4 |
| PLA2G16 | phospholipase A2, group XVI | -2.41 |
| PLAG1 | pleiomorphic adenoma gene 1 | -2.41 |
| SERPINE1 | serpin peptidase inhibitor, clade E member 1 | -2.41 |
| ABCG1 | ATP binding cassette subfamily G member 1 | -2.42 |
| PLAG1 | pleiomorphic adenoma gene 1 | -2.42 |
| CREBRF | CREB3 regulatory factor | -2.43 |
| FAM129A | family with sequence similarity 129, member A | -2.43 |
| SLCO4A1 | SLCO4A1 antisense RNA 1 | -2.43 |
| CREBRF | CREB3 regulatory factor | -2.43 |
| KCNN4 | potassium channel, calcium activated intermediate/small conductance subfamily N alpha, member 4 | -2.44 |
| RORA | RAR-related orphan receptor A | -2.44 |
| NCAM1 | neural cell adhesion molecule 1 | -2.44 |
| DHRS3 | dehydrogenase/reductase (SDR family) member 3 | -2.45 |
| SSBP2 | single-stranded DNA binding protein 2 | -2.45 |
| BTBD16 | BTB domain containing 16 | -2.45 |
| COL13A1 | collagen, type XIII, alpha 1 | -2.46 |
| NCAM1 | neural cell adhesion molecule 1 | -2.46 |
| ZBTB20 | zinc finger and BTB domain containing 20 | -2.47 |
| EGLN3 | egl-9 family hypoxia-inducible factor 3 | -2.47 |
| KLF9 | Kruppel-like factor 9 | -2.48 |
| TBC1D8B | TBC1 domain family, member 8B | -2.48 |
| N4BP2L1 | NEDD4 binding protein 2-like 1 | -2.48 |
| EFCAB3 | EF-hand calcium binding domain 3 | -2.49 |
| LILRA2 | leukocyte immunoglobulin-like receptor, subfamily A, member 2 | -2.51 |
| SIGLEC6 | sialic acid binding Ig-like lectin 6 | -2.51 |
| IZUMO4 | IZUMO family member 4 | -2.52 |
| COL13A1 | collagen, type XIII, alpha 1 | -2.54 |
| SORL1 | sortilin-related receptor, L(DLR class) A repeats containing | -2.54 |
| TNFSF9 | tumor necrosis factor (ligand) superfamily, member 9 | -2.55 |
| MTUS1 | microtubule associated tumor suppressor 1 | -2.57 |
| PDGFA | platelet-derived growth factor alpha polypeptide | -2.58 |
| ARRDC3 | arrestin domain containing 3 | -2.59 |
| LIMCH1 | LIM and calponin homology domains 1 | -2.59 |
| DDIT4 | DNA damage inducible transcript 4 | -2.59 |
| MTUS1 | microtubule associated tumor suppressor 1 | -2.6 |
| LIMCH1 | LIM and calponin homology domains 1 | -2.6 |
| TMPRSS3 | transmembrane protease, serine 3 | -2.6 |
| TPM2 | tropomyosin 2 (beta) | -2.6 |
| GMFG | glia maturation factor, gamma | -2.61 |
| ITGA10 | integrin alpha 10 | -2.61 |
| CASP4 | caspase 4 | -2.62 |
| KCNMA1 | potassium channel, calcium activated large conductance subfamily M alpha, member 1 | -2.63 |
| SLC16A6 | solute carrier family 16, member 6 | -2.65 |
| CAMK2N1 | calcium-dependent protein kinase II inhibitor 1 | -2.66 |
| SYTL3 | synaptotagmin-like 3 | -2.66 |
| EMP1 | epithelial membrane protein 1 | -2.66 |
| LIMCH1 | LIM and calponin homology domains 1 | -2.67 |
| PLOD2 | procollagen-lysine, 2-oxoglutarate 5-dioxygenase 2 | -2.68 |
| SLC16A6 | solute carrier family 16, member 6 | -2.7 |
| BCL6 | B-cell CLL/lymphoma 6 | -2.71 |
| RNASE4 | ribonuclease, RNase A family, 4 | -2.72 |
| RNASE4 | ribonuclease, RNase A family, 4 | -2.72 |
| DSC2 | desmocollin 2 | -2.74 |
| LIMCH1 | LIM and calponin homology domains 1 | -2.75 |
| SERPINE1 | serpin peptidase inhibitor, clade E (nexin, plasminogen activator inhibitor type 1), member 1 | -2.76 |
| NDRG1 | N-myc downstream regulated 1 | -2.76 |
| AHNAK2 | AHNAK nucleoprotein 2 | -2.77 |
| TPM2 | tropomyosin 2 (beta) | -2.78 |
| SLC16A4 | solute carrier family 16, member 4 | -2.79 |
| MTUS1 | microtubule associated tumor suppressor 1 | -2.8 |
| NDRG1 | N-myc downstream regulated 1 | -2.8 |
| NDRG1 | N-myc downstream regulated 1 | -2.8 |
| SATB1 | SATB homeobox 1 | -2.8 |
| TIMP3 | TIMP metallopeptidase inhibitor 3 | -2.81 |
| SLC16A6 | solute carrier family 16, member 6 | -2.81 |
| UBASH3B | ubiquitin associated and SH3 domain containing B | -2.82 |
| NDRG1 | N-myc downstream regulated 1 | -2.82 |
| TFF2 | trefoil factor 2 | -2.83 |
| TXNIP | thioredoxin interacting protein | -2.83 |
| FOSL2 | FOS-like antigen 2 | -2.83 |
| LOXL2 | lysyl oxidase-like 2 | -2.84 |
| TSC22D3 | TSC22 domain family, member 3 | -2.86 |
| MT1X | metallothionein 1X | -2.86 |
| SPRED1 | sprouty-related, EVH1 domain containing 1 | -2.86 |
| SORL1 | sortilin-related receptor, L (DLR class) A repeats containing | -2.87 |
| WFDC3 | WAP four-disulfide core domain 3 | -2.9 |
| TXNIP | thioredoxin interacting protein | -2.92 |
| MTUS1 | microtubule associated tumor suppressor 1 | -2.93 |
| LOC154761 | family with sequence similarity 115, member | -2.93 |
| CASP4 | caspase 4 | -2.96 |
| SPON1 | spondin 1 | -3.01 |
| TSC22D3 | TSC22 domain family, member 3 | -3.01 |
| LOXL2 | lysyl oxidase-like 2 | -3.02 |
| ARRDC3 | arrestin domain containing 3 | -3.03 |
| MT1X | metallothionein 1X | -3.04 |
| TXNIP | thioredoxin interacting protein | -3.07 |
| NDRG1 | N-myc downstream regulated 1 | -3.07 |
| CA9 | carbonic anhydrase IX | -3.08 |
| YPEL2 | yippee like 2 | -3.09 |
| CA9 | carbonic anhydrase IX | -3.1 |
| TXNIP | thioredoxin interacting protein | -3.1 |
| ERRFI1 | ERBB receptor feedback inhibitor 1 | -3.13 |
| NDRG1 | N-myc downstream regulated 1 | -3.15 |
| CA9 | carbonic anhydrase IX | -3.21 |
| SERPINE1 | serpin peptidase inhibitor, clade E， member 1 | -3.25 |
| CASP4 | caspase 4 | -3.27 |
| AHNAK2 | AHNAK nucleoprotein 2 | -3.28 |
| NRN1 | neuritin 1 | -3.36 |
| S100A9 | S100 calcium binding protein A9 | -3.37 |
| SCN3B | sodium channel, voltage gated, type III beta subunit | -3.4 |
| WFDC10B | WAP four-disulfide core domain 10B | -3.4 |
| LEMD1 | LEM domain containing 1 | -3.44 |
| SLC16A4 | solute carrier family 16, member 4 | -3.47 |
| NRN1 | neuritin 1 | -3.5 |
| SLC16A4 | solute carrier family 16, member 4 | -3.52 |
| ARRDC3 | arrestin domain containing 3 | -3.54 |
| SATB1 | SATB homeobox 1 | -3.55 |
| MTUS1 | microtubule associated tumor suppressor 1 | -3.56 |
| TXNIP | thioredoxin interacting protein | -3.6 |
| DDIT4L | DNA-damage-inducible transcript 4-like | -3.6 |
| TFF1 | trefoil factor 1 | -3.6 |
| SIGLEC6 | sialic acid binding Ig-like lectin 6 | -3.68 |
| CYP4F3 | cytochrome P450, family 4, subfamily F, polypeptide 3 | -3.68 |
| ITGA10 | integrin alpha 10 | -3.73 |
| CAPN8 | calpain 8 | -3.74 |
| IGFBP3 | insulin like growth factor binding protein 3 | -3.86 |
| IGFBP3 | insulin like growth factor binding protein 3 | -3.91 |
| SIGLEC6 | sialic acid binding Ig-like lectin 6 | -4.06 |
| ITGB8 | integrin beta 8 | -4.17 |
| PPP1R3B | protein phosphatase 1, regulatory subunit 3B | -4.69 |
